# Less is more! Low amount of *Fusobacterium nucleatum* supports macrophage-mediated trophoblast functions *in vitro*

**DOI:** 10.3389/fimmu.2024.1447190

**Published:** 2024-08-08

**Authors:** Rebekka Einenkel, Jens Ehrhardt, Marek Zygmunt, Damián Oscar Muzzio

**Affiliations:** Department of Gynecology and Obstetrics, University Medicine Greifswald, Greifswald, Germany

**Keywords:** placental microbiome, endometrial microbiome, decidual macrophages, implantation, extravillous trophoblast, *Fusobacterium nucleatum*, VEGF-A, HIF-1α

## Abstract

*F. nucleatum*, involved in carcinogenesis of colon carcinomas, has been described as part of the commensal flora of the female upper reproductive tract. Although its contribution to destructive inflammatory processes is well described, its role as commensal uterine bacteria has not been thoroughly investigated. Since carcinogenesis shares similar mechanisms with early pregnancy development (including proliferation, invasion, blood supply and the induction of tolerance), these mechanisms induced by *F. nucleatum* could play a role in early pregnancy. Additionally, implantation and placentation require a well-balanced immune activation, which might be suitably managed by the presence of a limited amount of bacteria or bacterial residues. We assessed the effect of inactivated *F. nucleatum* on macrophage-trophoblast interactions. Monocytic cells (THP-1) were polarized into M1, M2a or M2c macrophages by IFN-γ, IL-4 or TGF-β, respectively, and subsequently treated with inactivated fusobacteria (bacteria:macrophage ratio of 0.1 and 1). Direct effects on macrophages were assessed by viability assay, flow cytometry (antigen presentation molecules and cytokines), qPCR (cytokine expression), in-cell Western (HIF and P-NF-κB) and ELISA (VEGF secretion). The function of first trimester extravillous trophoblast cells (HTR-8/SVneo) in response to macrophage-conditioned medium was microscopically assessed by migration (scratch assay), invasion (sprouting assay) and tube formation. Underlying molecular changes were investigated by ELISA (VEGF secretion) and qPCR (matrix-degrading factors and regulators). Inflammation-primed macrophages (M1) as well as high bacterial amounts increased pro-inflammatory NF-κB expression and inflammatory responses. Subsequently, trophoblast functions were impaired. In contrast, low bacterial stimulation caused an increased HIF activation and subsequent VEGF-A secretion in M2c macrophages. Accordingly, there was an increase of trophoblast tube formation. Our results suggest that a low-mass endometrial/decidual microbiome can be tolerated and while it supports implantation and further pregnancy processes.

## 1 Introduction

Pregnancy requires local immune adaptation and support. While maternal immune cells must tolerate the (semi-)allogeneic fetus, the protection of mother and child against infections must not be impaired. Moreover, as trophoblast cells invade into the uterine wall, reaching spiral arteries and forming placental circulation, immune factors facilitate tissue remodeling processes (1, 2). In this context, Trophoblast cells are escorted by decidual macrophages and NK cells supporting their motility and invasive features (2, 3).

Pregnancy is accompanied with distinct immune phases. Implantation and placentation are, like every tissue remodeling process, dominated by rather inflammatory factors. As the placentation is completed the inflammatory phase is followed by a tolerogenic phase to maintain pregnancy while the fetus is growing (4). At the end of the third trimester, inflammatory responses induce labor (5).

Due to the fine-regulated nature of these processes, any disturbance of the immune balance can affect the wellbeing of mother and child. Exceeding pro-inflammatory reactions may cause rejection leading to miscarriage or preterm birth (6). Thus, any agent causing strong inflammatory responses is a threat to the pregnancy.

In this sense, the description of a placental microbiome challenged the understanding of the immune homeostasis during pregnancy (7). These studies were controversially discussed. However, the presence of a low abundant endometrial/decidual microbiome is generally assumed and considered to affect the implantation (8, 9). The detailed effect of the upper reproductive tract microbiome (URTM) on early pregnancy establishment remains largely unknown.

Among the described species, *F. nucleatum* was found in placental (7) and endometrial (10, 11) samples from healthy women. *F. nucleatum* is a well-described opportunist of the human oral cavity microbiome. In colon carcinoma, *F. nucleatum* is associated with tumorigenesis. There, *F. nucleatum* supports epithelial cell proliferation and migration, angiogenesis and the induction of tumor tolerance (12). Similar processes are essential for early pregnancy including invasion and migration of trophoblast cells, proliferation of trophoblast cells, angiogenesis on maternal and fetal side as well as the mediation of fetal tolerance. As comparable mechanisms drive both tumor progression and placentation processes, the presence of *F. nucleatum* at the URT might support placentation as described for tumors. There, trophoblast cells proliferate, invade into the decidua to anchor and build the placenta. Moreover, extravillous trophoblast cells invade into the spiral arteries to finally build the vessel perfusing the placenta later with maternal blood and thus also show angiogenic behavior, which is supported by decidual leukocytes (13).

In the oral cavity and during tumorigenesis, *F. nucleatum* affects not only tumor cells but also immune and endothelial cells. In murine macrophages, *F. nucleatum* increases the inflammatory cytokine response in a concentration-dependent manner (14). THP-1-derived macrophages increase the expression of chemokines due to the treatment with *F. nucleatum* driven by the activation of the transcription factor NF-κB (15). Even the BEVs (bacteria-derived extracellular vesicles) of *F. nucleatum* drive inflammatory responses by murine macrophages by the activation of NF-κB accompanied by the increased expression of TNF-α and iNOS. All of these studies examined the idea of an infection with suitably high MOIs (multiplicity of infection; bacteria:cell ratio) of 10 or 20 and higher. However, the URTM is characterized by its low abundance. Barely any studies address the effect of low abundant bacteria.

In inflammatory responses, NF-κB is a key transcription factor. It is induced by various signals including agonists of TLRs such as bacterial stimulants. Subsequently, NF-κB regulates a plethora of factors including mediators as cytokines for defense and cell recruitment and surface markers as antigen presentation molecules regulating adaptive immune responses.

During implantation, cytokines regulated by NF-κB are necessary to support invasion and the differentiation of trophoblasts (16). However, an excessive activation of NF-κB can lead to detrimental effects as seen in infections. This raises the question whether a microbiome with low abundance could stimulate immune responses, such as NF-κB activity, enough to facilitate implantation, but avoiding becoming a threat to the gestation by excessive activation.

Here, we studied the effect of inactivated, low abundant *F. nucleatum* on macrophages and its effect on trophoblast functions.

## 2 Materials and methods

### 2.1 Cell culture

Monocytic cell line THP-1 (ATCC, USA) cultured in RPMI1640 supplemented with 10% FBS, 1% Penicillin/Streptomycin (all from PAN-Biotech GmbH, Germany) and 50 µM β-mercaptoethanol (Sigma-Aldrich, USA) were differentiated into M0 macrophages with 10 ng/mL phorbol 12-myristate 13-acetate (PMA; Merck, Germany) for 48 h. 5 ×10^5^ cells/mL were used. M1 macrophages were differentiated by 50 ng/mL IFN-γ, M2a macrophages with 20 ng/mL IL-4 and M2c macrophages with 20 ng/mL TGF-β (all cytokines: R&D Systems, USA) for 24 h. Macrophages were treated with inactivated *Fusobacterium nucleatum* in a bacteria:macrophage ratio of 0.1 and 1.

Extravillous trophoblast cell line HTR-8/SVneo (ATCC, USA) were cultured in RPMI1640 supplemented with 10% FBS and 1% Penicillin/Streptomycin. For migration, invasion and tube formation assay, culture medium supplemented with charcoal hormone-depleted FBS was used.

### 2.2 Bacteria culture and inactivation

Fusobacteria were kindly provided by Elsa Baufeld (Friedrich Loeffler-Institut für Medizinische Mikrobiologie, University Medicine Greifswald). Bacteria were cultured on agar plates containing 5% sheep blood in GasPak systems (BD, USA). For inactivation bacteria were incubated under normoxic conditions (21% O_2_) for 24 h. Inactivated bacteria were washed and stored in PBS (Merck, Germany) at 4°C (17).

### 2.3 Molecular biology

#### 2.3.1 qPCR

Reverse transcription and quantitative PCR was performed as described before (18). RNA was isolated with peqGOLD TriFast (VWR, USA) and transcribed with High-Capacity cDNA Reverse Kit (Thermo Fisher Scientific, USA) following manufacturer’s instructions. Primer pairs were designed to span at least one exon-exon junction (see **Table 1**). Sequences of IGF-2 (19), MMP-9 (20), TIMP-1 and -2 (21) primer were used as described before.

**Table 1:** Primer sequences.

Target Transcript	Primer Orientation	Primer Sequence
*HPRT1*	forward	5′-TTTGCTTTCCTTGGTCAGGC-3′
	reverse	5′-TCAAATCCAACAAAGTCTGGCTT-3′
*CD40*	forward	5′-ACCCTTGGACAAGCTGTGAGAC-3′
	reverse	5′-TTTGATAAAGACCAGCACCAAGAG-3′
*CD80*	forward	5′-GGGCACATACGAGTGTGTTGTT-3′
	reverse	5′-CAGCTTTGACTGATAACGTCACTTC-3′
*HLADRA*	forward	5′-ACTATACTCCGATCACCAATGTACCTC -3′
	reverse	5′-AAGACTGTCTCTGACACTCCTGTGG-3′
*IGF2*	forward	5′-CCGGCTTCCAGACACCAAT-3′
	reverse	5′-GGCCAAGAAGGTGAGAAGCA-3′
*IL6*	forward	5′-TGGCAGAAAACAACCTGAACCT-3′
	reverse	5′-ACCAGGCAAGTCTCCTCATTGA-3′
*CXCL8*	forward	5′-TCTTGGCAGCCTTCCTGATT-3′
	reverse	5′-TTAGCACTCCTTGGCAAAACTG-3′
*MMP2*	forward	5’-TTGATGGCATCGCTCAGATC-3’
	reverse	5’-TCACAGTCCGCCAAATGAAC-3’
*MMP9*	forward	5′-CCCTGGAGACCTGAGAACCAAT-3′
	reverse	5′-CCCGAGTGTAACCATAGCGGTA-3′
*TIMP1*	forward	5′‐CAATTCCGACCTCGTCATCAG‐3′
	reverse	5′‐CGCTGGTATAAGGTGGTCTGGT‐3′
*TIMP2*	forward	5′‐GAAACGACATTTATGGCAACCC‐3′
	reverse	5′‐TTCTCAGGCCCTTTGAACATCT‐3′

### 2.4 Immunological methods

#### 2.4.1 Flow cytometry

For flow cytometry analysis, macrophages were incubated with monensin 5 h prior staining. Live/dead cells were distinguished though staining with Fixable Viability Dye (Thermo Fisher Scientific, USA), incubated for 30 min at 4°C in the dark. Extracellular staining was performed for 30 min at 4°C in the dark. Applied antibodies: CD40 (FGK45.5; Miltenyi Biotec, Germany), CD80 (L307.4; BD, USA) and HLA-DR (AC122; Miltenyi Biotec, Germany). Cells were fixed with Cytofix/Cytoperm (BD, USA) for 20 min in the dark. Cells were measured by FACSCanto. As gating controls, negative and “fluorescence minus one” (FMO) stained samples were included.

#### 2.4.2 ELISA

Conditioned media were centrifuged at 1000 ×*g* for 5 min and stored at -80°C. VEGFA ELISA (R&D, USA) was performed based on the manufacturer’s instructions. Signal was measured with FLUOstar OPTIMA Microplate Reader (BMG Labtech, Germany).

#### 2.4.3 In-cell-western

Macrophages cultured in 96-well plates were fixed with 3.7% PFA (Carl Roth, Germany) for 20 min and permeabilized with ice cold methanol (Merck, Germany) for 30 min. After blocking with Intercept Blocking Buffer (LI-COR, USA), cells were incubated with primary antibody in Intercept Blocking Buffer containing 0.2% Tween-20 (Sigma-Aldrich, USA) overnight at 4°C. Applied antibodies: P(Ser536)-NF-κB (93H1; Cell Signaling Technology, USA) or HIF-1α (R&D systems, USA). Cells were washed with 0.1% Tween-20 in PBS four times and incubated with secondary antibody (anti-rabbit-IR-Dye 800CW-conjugated or anti-goat-IR-Dye 800CW-conjugated; LI-COR, USA) and DRAQ5 (Cell Signaling Technology, USA) for DNA staining for 60 min at room temperature in the dark. Fluorescent signal was captured with LI-COR Odyssey imager.

### 2.5 Trophoblast behavioral assays

#### 2.5.1 Scratch assay

The scratch was performed in confluent HTR-8/SVneo with a pipette tip. During migration assay trophoblast cells were stimulated with 20% macrophage-conditioned medium. Regrowth was microscopically assessed by Observer Z.1 microscope (Carl Zeiss Microscopy, Germany) with an incubation system. During live cell imaging, every hour two images per well were taken for 24 h. Cell-free area was measured by ImageJ Wound Healing Tool.

#### 2.5.2 Sprouting assay

Trophoblast spheroids were obtained by the cultivation of 10³ cells per well in an U-bottom well plate (Sarstedt, Germany) in culture medium containing 5% methyl cellulose (Sigma-Aldrich, USA). Spheroids were embedded in 10 mg/mL growth factor-reduced matrigel (Corning, USA). After polymerization, culture medium containing 20% macrophage-conditioned medium was added. Spheroids were visualized by Observer Z.1 microscope at 0 h, 24 h and 48 h after treatment.

#### 2.5.3 Tube formation assay

The lower chamber of µ-Slides for angiogenesis (ibidi, Germany) were coated with 5 mg/mL matrigel. For 2D tube formation, 8 ×10³ HTR-8/SVneo cells were seeded on the matrigel layer in the presence of 20% macrophage-conditioned medium.

### 2.6 Statistical analyses

Data was analyzed using GraphPad Prism 8 (GraphPad Software, USA). Data was assumed normally distributed. Statistical analyses were applied as indicated in the figure legends. P-value ≤ 0.05 were assume statistical significant. The effect of LPS was analyzed by Student’s *t*-Test. The effect of *F. nucleatum* was analyzed by Repeated Measures ANOVA with Tukey’s posttest.

## 3 Results

### 3.1 Low mass bacteria are a mild inducer of macrophage pro-inflammatory activation

**Figure 1:**
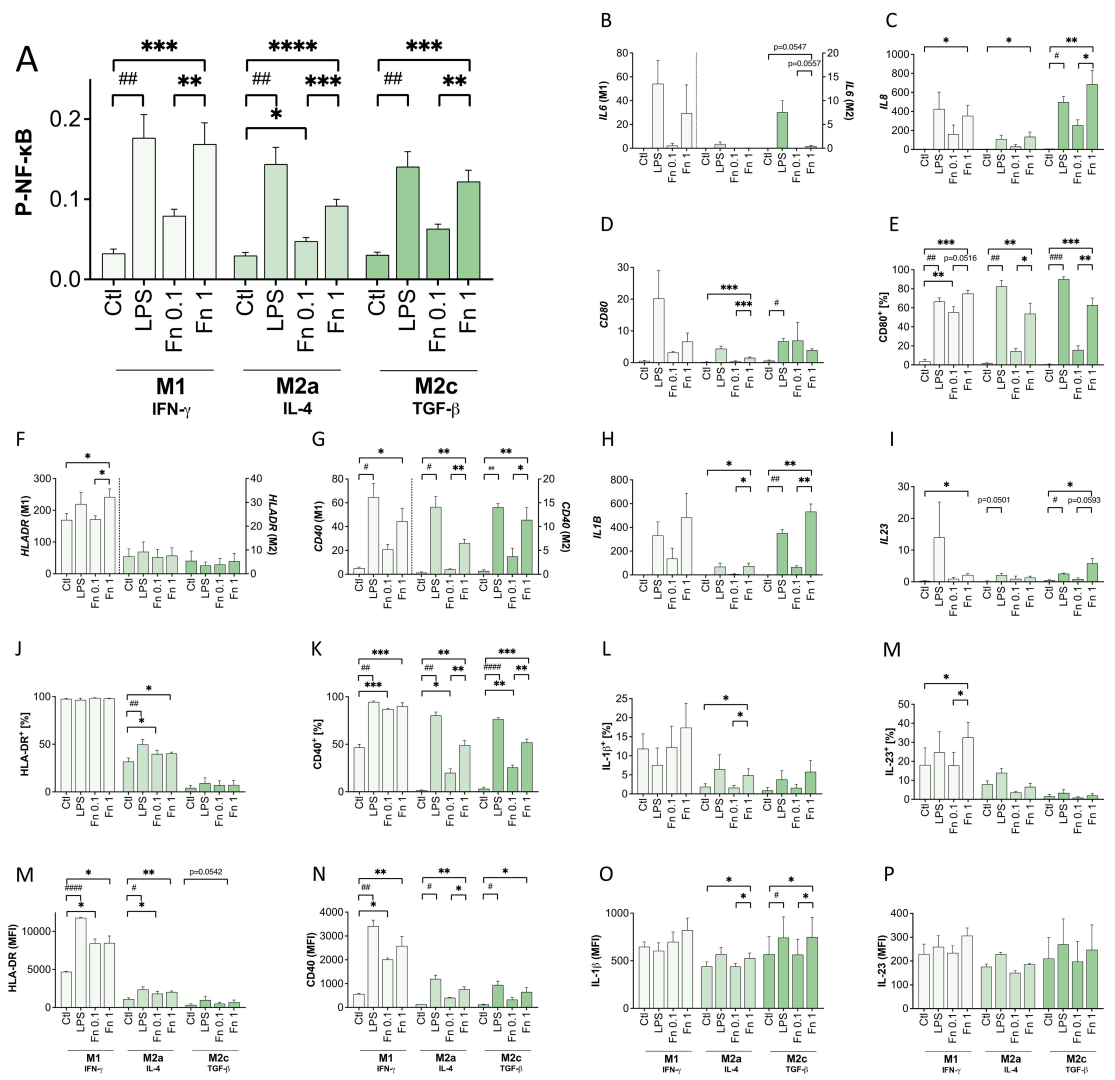
Low bacterial stimulation induces P-NF-κB and NF-κB-regulated factors in a dose-dependent manner. Differentiated THP-1-derived macrophages were treated with 10 ng/mL LPS or inactivated *F*. *nucleatum* (bacteria:cell ratio of 0.1 or 1) for 24 h. **(A)** The expression of phosphorylated NF-κB was assessed by an In-Cell-Western assay normalized to the DRAQ5 signal. n=5 in quadruplicates. **(B–D, F–I)** The expression of *IL6*, *IL8*, *HLADR*, *CD40*, *CD80*, *IL1B* and *IL23* transcripts was assessed in relation to the *HPRT1* expression. n=3 in duplicates. **(E, J–P)** The surface expression of HLA-DR, CD40, CD80 and the intracellular expression of IL-1β and IL-23 were additionally assessed by flow cytometry. The proportion of positive cells as well as the MFI (mean fluorescence intensity) were measured. n=3. **(A–P)** Bars show mean with SEM. Expression changes were analyzed by Student’s *t*-Test for the effect of LPS (# *p*-value ≤ 0.05; ## *p*-value ≤ 0.01; ### *p*-value ≤ 0.001) or with Repeated Measures ANOVA with Tukey’s posttest for the effect of *F*. *nucleatum* (* *p*-value ≤ 0.05; ** *p*-value ≤ 0.01; *** *p*-value ≤ 0.001; **** *p*-value ≤ 0.0001). Borderline *p*-values under 0.06 are shown.

Phosphorylated NF-κB was assessed by In-Cell-Western assay. As expected, bacterial treatment increased the activated form of NF-κB (see **Figure 1**). *F. nucleatum* increased NF-κB activation dose-dependently resulting in significant differences between lower and higher concentration in all macrophage subtypes (M1: p=0.0054; M2a: p=0.0001; M2c: p=0.0022).

Accordingly, NF-κB-regulated cytokines, such as IL-1β, IL-6, IL-8 and IL-23, were expressed in a similar pattern on RNA and protein level increasing with the stimulatory dosage. In parallel to the cytokine expression, antigen presentation-related surface molecules were induced after bacterial treatment. Again, the lower *F. nucleatum* concentration led to a slight increase and was further increased by the higher concentration especially in co-stimulatory molecules CD40 and CD80. The MHCII molecule HLA-DR was also induced by bacterial stimulation, but subtype-specific differences prevailed. M1 macrophages showed the strongest HLA-DR expression, whereas M2a macrophages showed a medium expression and M2c macrophages the lowest HLA-DR expression on RNA and surface protein level.

Both concentrations of *F. nucleatum* activated all macrophage subtypes, which displayed a stronger response to the higher concentration.

### 3.2 Bacterial presence affects macrophage-regulated trophoblast functions

In general, decidual macrophages support invasive trophoblast cell behavior. As bacterial presence activated macrophages in this study even in very low abundance, the effect on the macrophage-regulated trophoblast functions was assessed. Conditioned medium of bacteria-stimulated macrophages was used to treat cells of the HTR-8/SVneo trophoblast cell line. Typical extravillous trophoblast functions, such as migration, invasion and tube formation were assessed.

#### 3.2.1 Trophoblast migration

**Figure 2:**
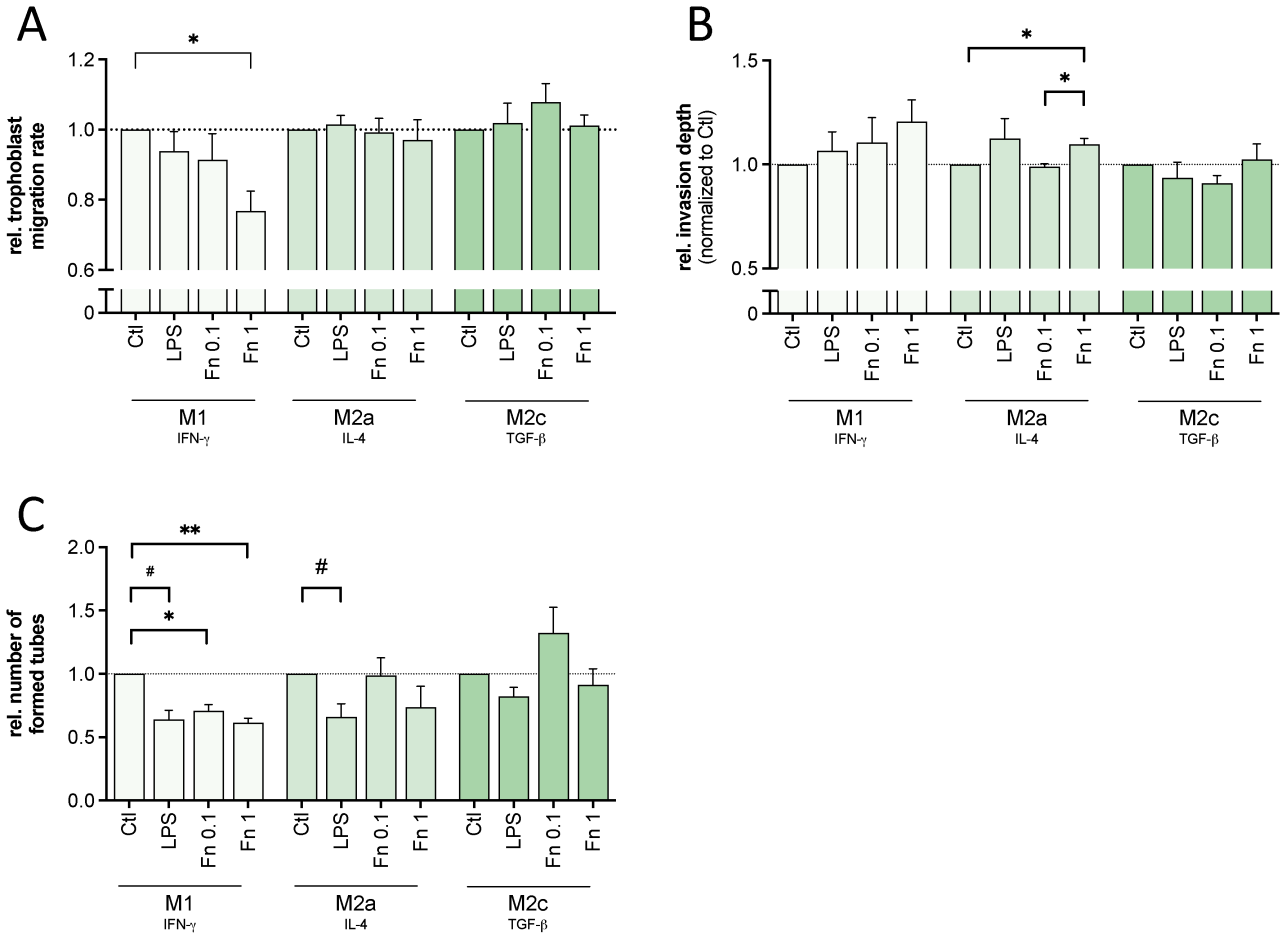
Macrophages treated with low bacterial stimulation affected trophoblast behavior. Conditioned medium of differentiated THP-1-derived macrophages treated with 10 ng/mL LPS or inactivated *F*. *nucleatum* (bacteria:cell ratio of 0.1 or 1) was used to treat trophoblastic cells (HTR-8/SVneo). Trophoblast migration, invasion and tube formation capacity was assessed microscopically. **(A)** A scratch assay using trophoblast cells was conducted. The cell-free area was measured every hour to calculate mean velocity. The values were normalized to untreated control. n=4 in triplicates. **(B)** Trophoblast cells were cultured as spheroids and embedded in a 3D matrix using Matrigel. The change in the size of the spheroid including its sprouts between 0 to 48 h were normalized to the untreated control. n=4-5 with 3-7 replicates. **(C)** Trophoblast cells were cultured on top of Matrigel. The number of formed 2D tubes was assessed after 6 h and normalized to the untreated control. n=4 in triplicates. **(A–C)** Bars show mean with SEM. Expression changes were analyzed by Student’s *t*-Test for the effect of LPS (# *p*-value ≤ 0.05) or with Repeated Measures ANOVA with Tukey’s posttest for the effect of *F*. *nucleatum* (* *p*-value ≤ 0.05; ** *p*-value ≤ 0.01). Borderline *p*-values under 0.06 are shown.

The migratory function of trophoblast cells was affected by the bacterial treatment of macrophages (see **Figure 2A**). M1 macrophages treated with *F. nucleatum* impaired trophoblast cell migration with rising concentration (M1+Fn1: p=0.0137). However, there was no significant effect mediated by bacteria-treated M2a or M2c macrophages compared to untreated M2a or M2c macrophages. LPS alone did not change migration rate by any macrophage subtype significantly.

#### 3.2.2 Trophoblast invasion

Throughout implantation and placentation invasive trophoblast behavior is required. For this, trophoblast cells destruct extracellular matrix components activated by immune factors, like IL-6 and IL-8.

M1 or M2c macrophage-mediated invasive trophoblast cell behavior was not significantly changed by bacterial stimulation (see **Figure 2B**). In contrast, rising bacterial concentration increased M2a macrophage-mediated trophoblast cell invasion significantly with both *F. nucleatum* (M2a+Fn0.1: p=0.0289; M2a+Fn1: p=0.0177). Again, LPS alone did not change any macrophage-mediated invasive trophoblast cell function.

In fact, bacterial treatment affected macrophage-mediated expression of invasion-related factors (*MMP2*, *MMP9*, *TIMP1*, *TIMP2* and *IGF2*) in trophoblast cells. A single factor explaining the differences between M1, M2a or M2c was not found (see **Supplementary Figure S3**).

#### 3.2.3 Trophoblast tube formation

Assembling the placental blood supply, trophoblast cells invade into spiral arteries and form the lumen for maternal blood perfusion. Therefore, cell organization and tube formation abilities are required.

Bacterial treatment as well as LPS led to a significant decrease of M1 macrophage-mediated trophoblast cell tube formation (see **Figure 2C**). Similarly, LPS-treatment of M2a macrophages significantly decreased trophoblast cell tube formation. However, the lower concentration of *F. nucleatum* tendentially increased trophoblast cell tube formation by M2c macrophages. Although not statistically significant, all 4 experimental replicates (consisting of 3 technical replicates) showed an increase compared to the untreated macrophage control, supporting trophoblast function. A similar difference compared to M2c macrophages treated with the high bacterial amount (Fn1) was observed.

In conclusion, activated M1 macrophages showed negative effects on trophoblast migration and tube formation. Activated M2a macrophages showed both improving and impeding effects. Only activated M2c macrophages showed no negative but tendential positive effects.

### 3.3 Low bacterial stimulation activates the HIF-VEGF axis in M2c macrophages

Since tube formation is driven by factors, like VEGF (see **Supplementary Figure S4**), the expression of VEGF-A in activated macrophages was assessed by qPCR and ELISA.

**Figure 3:**
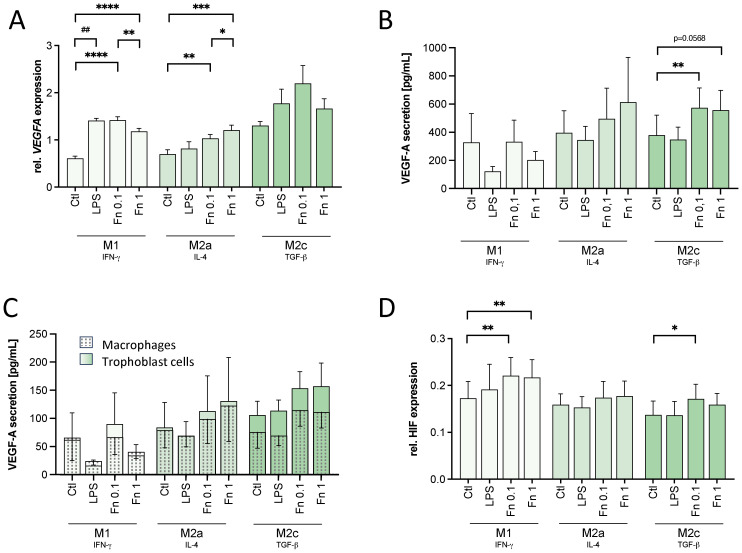
VEGF and HIF-1α expression is affected by low bacterial stimulation in macrophages. THP-1-derived macrophages were treated with 10 ng/mL LPS or inactivated *F*. *nucleatum* (bacteria:cell ratio of 0.1 or 1) for 24 h. VEGF and HIF-1α expression was assessed. **(A)** *VEGFA* expression on a transcriptional level was assessed in treated macrophages by qPCR and normalized to the expression of *HPRT1*. n=3 in duplicates. **(B, C)** The secretion of VEGF-A by treated macrophages **(B)** and trophoblast cells cultured in the presence of macrophage-conditioned medium **(C)** was assessed by ELISA. n=4. **(D)** Changes in the expression of HIF-1α were assessed by In-Cell Western assay normalized to the DRAQ5 signal. n=5 in quadruplicates. **(A–D)** Bars show mean with SEM. Expression changes were analyzed by Student’s *t*-Test for the effect of LPS (## *p*-value ≤ 0.01) or with Repeated Measures ANOVA with Tukey’s posttest for the effect of *F. nucleatum* (* *p*-value ≤ 0.05; ** *p*-value ≤ 0.01; *** *p*-value ≤ 0.001; **** *p*-value ≤ 0.0001). Borderline *p*-values under 0.06 are shown.

To evaluate the direct role of bacteria on macrophage VEGF-A expression, the transcription and secretion of VEGF-A was assessed. Bacterial treatment of macrophages led to an increase of *VEGFA* transcription in all macrophages by both *F. nucleatum* concentrations compared to the untreated control (see **Figure 3A**). In M2c macrophages, the lower *F. nucleatum* concentration increased *VEGFA* expression even stronger than the higher *F. nucleatum* stimulation although not statistically significant (see **Figure 3A**).

Assessed by ELISA, *F. nucleatum* induced VEGF-A secretion as well in macrophages (see **Figure 3B**). In M2a (p=0.0538) and M2c (p=0.0591) macrophages, VEGF-A secretion was tendentially increased by the higher bacterial concentration. In M2c macrophages, the lower concentration of *F. nucleatum* significantly increased VEGF-A secretion. LPS had no significant effect on VEGF-A secretion in any macrophage subtype.

No significant differences were found in VEGF-A secretion of trophoblast cells treated with macrophage-conditioned medium (see **Figure 3C**).

As *VEGFA* transcription is modulated by the transcription factor HIF-1α, its expression was assessed by In-Cell-Western assay. In M1 macrophages, HIF-1α expression was significantly increased after treatment with both *F. nucleatum* concentrations confirming the effect seen on *VEGFA* expression (see **Figure 3D**). In M2a macrophages, no significant difference was seen in HIF-1α expression (see **Figure 3D**). In M2c macrophages, instead, the lower concentration of *F. nucleatum* significantly increased the HIF-1α signal (see **Figure 3D**). The higher *F. nucleatum* concentration did not lead to a significant change in the HIF-1α signal of M2c macrophage (see **Figure 3D**). Thus, the expression of HIF-1α confirmed the expression and secretion pattern of VEGF-A.

In conclusion, the mild bacterial stimulation was enough to activate the macrophages, but only to a limited, significantly lower degree compared to high bacterial amounts. However, the mild bacterial stimulation by *F. nucleatum* was at least as potent as the higher bacterial amounts to stabilize HIF-1α. In M2c macrophages, the mild bacterial stimulation led to an even higher HIF-1α signal and VEGF-A secretion than the high bacterial stimulation.

## 4 Discussion

An increasing body of evidence indicates that the upper reproductive tract microbiome (URTM) has an impact on female fertility and pregnancy outcome. Various analyses found a correlation between the URTM with fertility and pregnancy outcomes. Patients with recurrent implantation failure (RIF) show altered URTM characteristics concerning the diversity, *Lactobacillus* dominance and presence of certain bacteria [e.g. *Streptococcus*, *Dialister* and *Prevotella* (22); *Gardnerella*, *Burkholderia*, *Atopobium*, *Delftia* (10, 23)]. Similarly in IVF patients, the presence [H_2_O_2_-producing *Lactobacillus* (24)*, Lactobacillus* spp.*, Anaerobacillus* spp.*, Burkholderia* spp.*, and Gardnerella* spp. (25)] or absence [*Streptococcus viridans, Atopobium, Bifidobacterium, Chryseobacterium, Gardnerella, Haemophilus, Klebsiella, Neisseria, Staphylococcus, and Streptococcus* (24, 26)] of certain bacteria correlates with the pregnancy rate or live birth rate. However, neither the causal link, nor the interaction between the URTM, endometrial cells and trophoblast cells are adequately characterized.

Both implantation and placentation take place under a fine-regulated inflammatory milieu. Immune activation and the release of pro-inflammatory mediators facilitate tissue remodeling, while anti-inflammatory mechanisms warrant fetal tolerance. When pathogens colonize the URT during infections, a vast inflammatory stimulus, represents an evident threat to the ongoing gestation. However, in the absence of infections, the URTM is characterized by low abundance of bacteria. Thus, a low mass microbiome might mildly stimulate immune cells to support early pregnancy processes without causing a destructive immune response. The few available studies addressing the effect of low-abundant bacteria in the URT suggest that a mild stimulation might have a positive effect on pregnancy outcome (27, 28).

In cows, an intra-uterine infusion of low dose LPS improves fertilization success. In contrast, the treatment with a high LPS amount does not show any improving effects. After infusion with the supporting low LPS concentration, there is no significant leukocyte influx into uterine tissue, indicating that only a locally limited immune activation takes place (29). This mild activation of immune cells supports implantation processes. On the one hand, trophoblast cells might be affected directly. In human trophoblast cells, a moderate stimulation with *F. nucleatum* (Fn0.1-Fn1) induces invasion along with the expression of matrix-modulating factors (27). In contrast to low bacterial stimulation, a higher bacterial amount rather shows detrimental effects on trophoblast cells (27, 28). Here, although the consecutive dilution might have confronted the trophoblast cells with Fn0.6 and Fn0.06, the effects on trophoblast behavior differed and were depended on the macrophage differentiation, leading to assume a greater effect of the factors secreted by macrophages.

On the other hand, bacteria might act through leukocytes, which are especially equipped with pattern recognition receptors (PRRs). Thereby, decidual immune cells affect trophoblast cells and vice versa. In trophoblast-educated monocytes, contradictory effects of a mild vs. clearly inflammatory stimuli by LPS were described. Low stimulation with 0.1 mg/mL LPS decreases the expression of the pro-inflammatory factors GRO-*α*, MCP-1, MIP-1β, RANTES, IL-1β, IL-6 and TNF-*α* by trophoblast-educated monocytes reducing the inflammatory potential, whereas 10 mg/mL LPS increases these factors (30).

While the aforementioned studies indicated that low doses of *E. coli*-derived LPS could have beneficial effects, we found that 10 ng/mL LPS had no effect or was even detrimental. Given that one bacterium can contain up to 50 fg of LPS (31), a comparable *E. coli* concentration at a ratio of 0.1 would have contained 2.5 ng/mL LPS. Due to the spatial distribution, LPS on the surface of a bacterium is barely comparable to free LPS in the culture medium. Thus, the used LPS concentration might still be too high rather inducing type 1 inflammatory responses even in M2-primed macrophages. As further virulence factors might also influence macrophage function, this work should not be oversimplified to the role of LPS-TLR4 interactions. Subsequent projects could address the relevance of different macrophage-bacteria interaction in the promotion of angiogenesis more precisely.

We showed that *in vitro*-generated macrophages react to low concentration of bacteria by displaying a moderate inflammatory phenotype and considerable VEGF-A release. The effect on trophoblasts depended on both the bacterial amount and also on the macrophage subtype. Since the decisive factor of macrophage education by trophoblast cells is TGF-β, M2c macrophages reflect the trophoblast-educated macrophages most strongly (32). In these cells, the small amount of bacteria was not only able to activate the macrophages. The stimulation of M2c macrophages with the low amount of *F. nucleatum* also resulted in an increased *HIF1A* expression and VEGF-A expression and secretion compared to the untreated control but, most interestingly, also compared to the cells stimulated with high amounts of *F. nucleatum*.

HIF-1α can be activated by oxygen tension, but also independently of the oxygen concentration by factors including hormones, cytokines and growth factors (33), which are also expressed at the feto-maternal interface. Also, various bacterial components can activate HIF-1α, including adhesins, LPS, siderophores and toxins (34). The transcription factor HIF-1α regulates the transcription of over 70 target genes directly including VEGF (35). Since the placenta develops in a low oxygen environment, HIF is a key regulator in implantation and placentation (36). We showed that a mild bacterial stimulus, such as the low abundant URTM, can also contribute to the expression or stabilization of HIF-1α leading to VEGF secretion. VEGF supports angiogenesis, which is massively required during placentation.

Moreover, VEGF also affects macrophages directly. Autocrine VEGF causes the upregulation of PD-L1 in murine M2 macrophages (37). PD-L1 is also expressed by human decidual macrophages. There, the interaction of PD-L1 on decidual macrophages with PD-1 on T cells suppresses the release of IFN-γ contributing to a tolerogenic milieu (38). This means that a mild bacterial stimulation could contribute to a fetal tolerance.

A macrophage subtype, which is characterized by its secretion of VEGF, encompasses M2d macrophages. These are also known as tumor-associated macrophages (TAMs) (39). Tumor development and processes of early pregnancy share certain mechanisms. In both, cells show proliferation, invasiveness, angiogenesis and mediate tolerance (40). *F. nucleatum* is also associated with colorectal cancer supporting tumor progression (41). Its presence might induce the TAMs in a similar way as shown supporting tumor development.

The majority of the studies addressing the URTM are based on sequencing methods that cannot determine bacteria viability. Recent culture-based omics studies (culturomics) depict differences between these and classical sequencing methods (42). Differences might be related to amplification of contaminants, dead bacteria or bacterial derived particles as extracellular vesicles (BEVs) (43). Dead bacteria or membrane components might still exert inflammatory or immunomodulatory effects, even in low concentrations. In order to replicate this possibility *in vitro*, all bacterial treatments were performed with inactivated bacteria in ratios of one bacterium per cell or even lower, one bacterium each ten cells. This allowed us to explore concentrations approximately 20-100 times lower that classical infection-methods and, as inactivated bacteria do not replicate, to strictly control bacteria to cell ratio. On the other hand, comparing living bacteria could imply adapting culture conditions according to their aerobic or anaerobic requirements, adding complexity to the analysis of cell function.

As it is known that an infection with *F. nucleatum* is a serious threat to the pregnancy, this study focused on a mild bacterial stimulation as expected by the low abundant URTM. Although no studies could determine with precision the exact number of bacteria present in the endometrial cavity, the findings of this study indicate that a ratio of as few as one bacterium per ten cells is sufficient to influence macrophage functionality. Testing of endometrial bacteria is already commercially available. However, the results are of limited significance as long as the interactions are not known. More research is needed to understand which core microbes at which abundance supports fertility, which changes are tolerable or may cause dysbiosis.

### 4.1 Outlook

The results of this work encourage further studies to determine possible mechanisms of immune regulation of decidual cells through bacteria. While many studies that aim to determine a “core” uterine microbiota are ongoing, they still face challenges and limitations of characterizing low-mass microbiota by molecular-based methods. The use of “culturomics” to previously enrich the sample before sequencing is a promising alternative which also permits the identification of live bacteria in uterine samples (42, 44). At the same time, it offers the possibility to use these patient-derived bacteria for further *in vitro* experiments.

The use of endometrial explants or endometrial organoids offer an interesting substitute to immortalized cell lines and permit the evaluation of material derived from fertile patients and patients with infertility (45). Therefore, possible therapeutic approaches based on microbiota composition can be tested.

### 4.2 Conclusion

**Figure 4:**
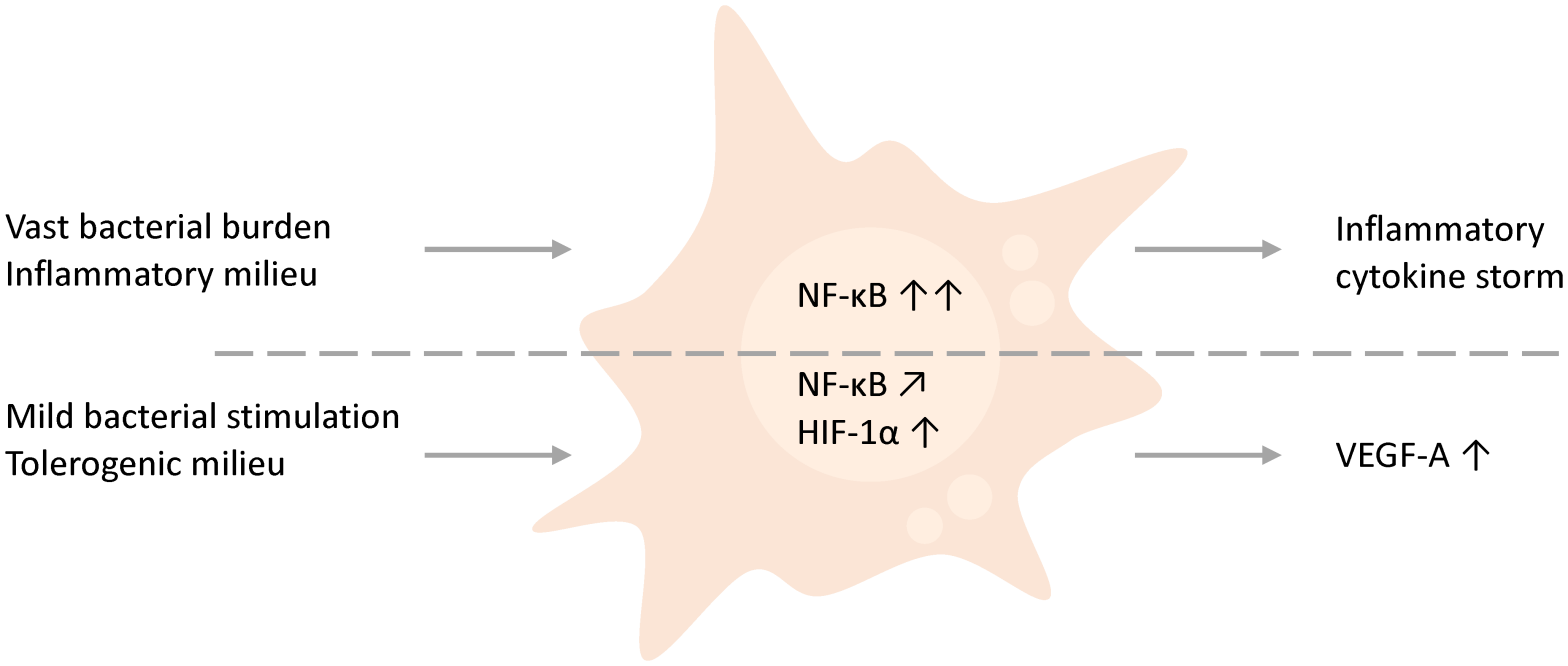
Schematic comparison and hypothesis. THP-1-derived macrophages, which either face higher bacterial amounts or are inflammation-primed by the presence of inflammatory factors such as IFN-γ, obstruct trophoblast behavior by the initiation of a cytokine storm. However, in contrast a mild stimulation of macrophages by low abundant bacteria or bacterial compounds in a general tolerogenic microenvironment, as found in early pregnancy, supports trophoblast functions and affects the environment by the secretion of VEGF.

The treatment of inflammatory-primed M1 macrophage with even small bacterial stimulation compromised trophoblast function. However, in tolerogenic M2c macrophages, low bacterial concentrations supported trophoblast tube formation. This effect correlated with significantly enhanced VEGF-A secretion and HIF-1α stabilization (**Figure 4**). Thus, a moderate stimulation by bacterial components can, in contrast to vast destructive immune activation, support implantation processes.

## Data availability statement

The raw data supporting the conclusions of this article will be made available by the authors, without undue reservation.

## Ethics statement

Ethical approval was not required for the studies on humans in accordance with the local legislation and institutional requirements because only commercially available established cell lines were used.

## Author contributions

RE: Conceptualization, Data curation, Formal analysis, Investigation, Methodology, Validation, Visualization, Writing – original draft, Writing – review & editing. JE: Investigation, Writing – review & editing. MZ: Funding acquisition, Resources, Supervision, Writing – review & editing. DM: Conceptualization, Methodology, Project administration, Supervision, Writing – review & editing.
