# Supplementary material for: Less is more! Low amount of Fusobacterium nucleatum supports macrophage-mediated trophoblast functions in vitro
Source: Front Immunol. 2024 Aug 8;15:1447190. doi: 10.3389/fimmu.2024.1447190 (PMC11338817; doi:10.3389/fimmu.2024.1447190)
Supplement: Supplementary file 1 [file DataSheet_1.docx]

Supplementary Material

Less is more! Low amount of *Fusobacterium nucleatum* supports macrophage-mediated trophoblast functions *in vitro*

Rebekka Einenkel, Jens Ehrhardt, Marek Zygmunt and Damián Muzzio*

*** Correspondence:**Dr. rer. med. Damián Muzzio
damian.muzzio@med.uni-greifswald.de

## Supplementary Figures

**Supplementary Figure 1.** **Macrophage viability and metabolic activity is maintained after bacterial treatment.** THP-1-derived macrophages were treated with 10 ng/mL LPS or inactivated *F. nucleatum* (bacteria:cell ratio of 0.1 or 1) for 24 h. Cellular viability and metabolic activity was assessed by CellTiterBlue turnover measured photometrically. n=4 in quadruplicates. Bars show mean with SEM. Expression changes were analyzed by Student's *t*-Test for the effect of LPS (# *p*-value ≤ 0.05; ## *p*-value ≤ 0.01; ### *p*-value ≤ 0.001) or with Repeated Measures ANOVA with Tukey’s posttest for the effect of *F. nucleatum* (* *p*-value ≤ 0.05; ** *p*-value ≤ 0.01; *** *p*-value ≤ 0.001). Borderline *p*-values under 0.06 are shown.


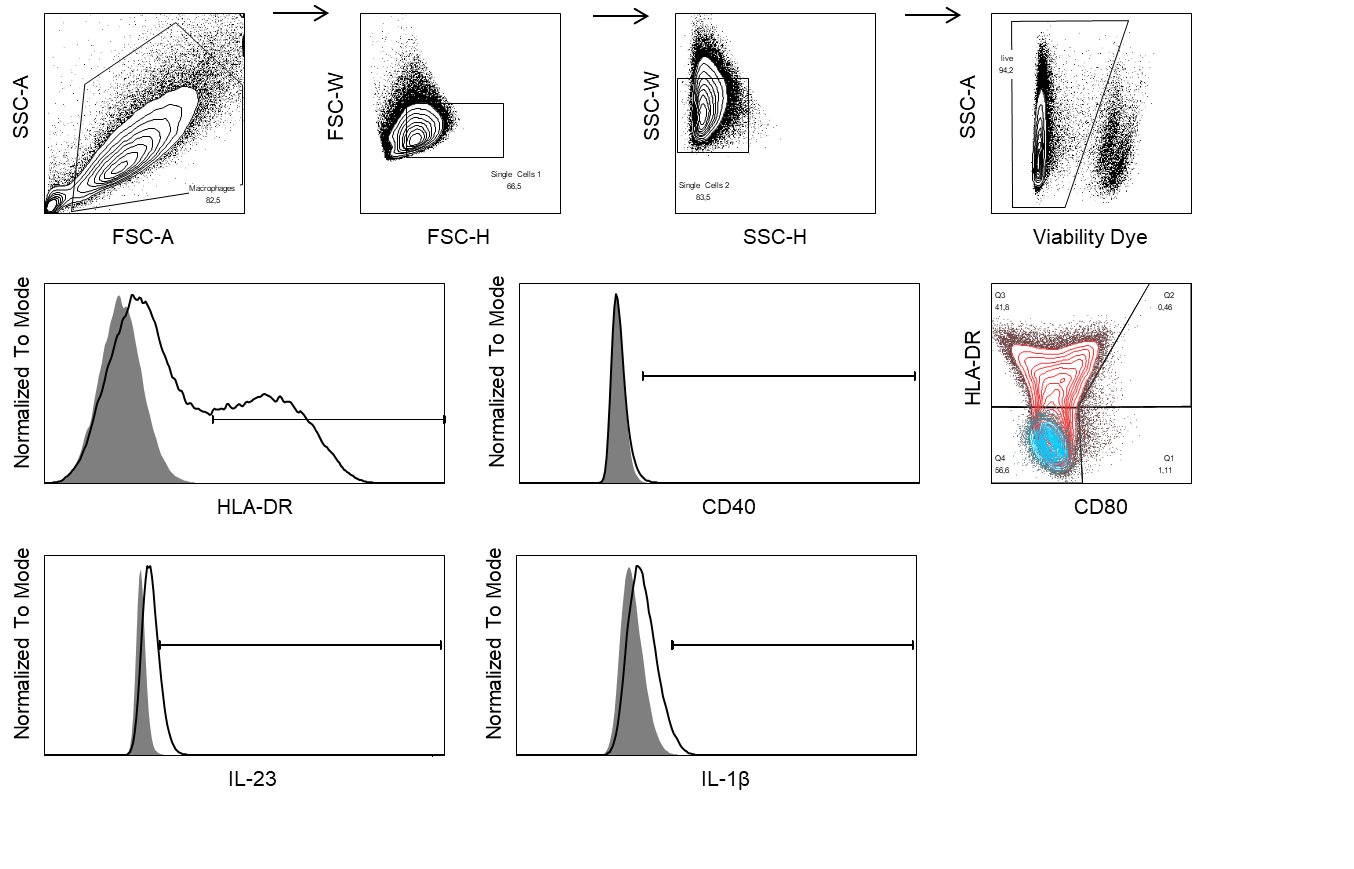


**Supplementary Figure 2. Gating strategy for flow cytometric analysis.** Representative Plots are shown for the gating strategy. In the histograms and plots, FMO (grey or blue) are shown in comparison to stained cells (white or red).

**Supplementary Figure 3. Expression of matrix-degenerating factors and their regulators by bacteria-treated macrophages.**The transcriptional expression of *TIMP1*, *TIMP2*, *MMP2*, *MMP9* and *IGF2* by THP-1-derived macrophages treated with 10 ng/mL LPS or inactivated *F. nucleatum* (bacteria:cell ratio of 0.1 or 1) for 24 h was assessed in comparison to the expression of *HPRT1* by qPCR. n=3 in duplicates. (**A-C**) Bars show mean with SEM. Expression changes were analyzed by Student's *t*-Test for the effect of LPS (# *p*-value ≤ 0.05) or with Repeated Measures ANOVA with Tukey’s posttest for the effect of *F. nucleatum* (* *p*-value ≤ 0.05). Borderline *p*-values under 0.06 are shown.


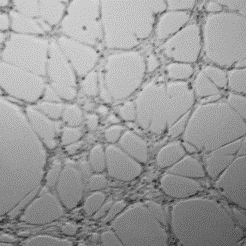

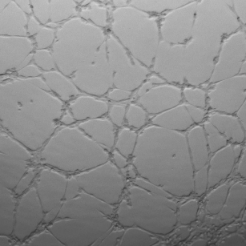


A

B

250 µm

+50 ng/mL VEGF

Untreated

**Supplementary Figure 4. Trophoblast tube formation in the presence and absence of VEGF.** HTR-8SV/neo trophoblastic cells were cultured on top of Matrigel matrix. The formation of 2D tubes was assessed microscopically in the (B) presence or (A) absence of 50 ng/mL recombinant human VEGF. Representative images are shown.
